# Derlin-1, as a Potential Early Predictive Biomarker for Nonresponse to Infliximab Treatment in Rheumatoid Arthritis, Is Related to Autophagy

**DOI:** 10.3389/fimmu.2021.795912

**Published:** 2022-01-03

**Authors:** Yongsong Cai, Ke Xu, Yirixiati Aihaiti, Zhijin Li, Qiling Yuan, Jing Xu, Haishi Zheng, Mingyi Yang, Bo Wang, Yanni Yang, Yin Yang, Peng Xu

**Affiliations:** ^1^ Department of Joint Surgery, Xi’an Honghui Hospital, Xi’an Jiaotong University Health Science Center, Xi’an, China; ^2^ Department of Neurosurgery, First Affiliated Hospital of the University of Science and Technology of China, Hefei, China; ^3^ Department of Biochemistry and Molecular Biology, School of Basic Medical Sciences, Xi’an Jiaotong University Health Science Center, Xi’an, China; ^4^ Department of Orthopaedics of the First Affiliated Hospital, Xi’an Jiaotong University Health Science Center, Xi’an, China; ^5^ Center for Translational Medicine, The First Affiliated Hospital of Xi’an Jiaotong University Health Science Center, Xi’an, China; ^6^ Department of Clinical Medicine of Traditional Chinese and Western Medicine, Shaanxi University of Traditional Chinese Medicine, Xianyang, China; ^7^ Department of Orthopaedics, Xi’an Central Hospital, Xi’an Jiaotong University Health Science Center, Xi’an, China

**Keywords:** rheumatoid arthritis, derlin-1, infliximab, predictive biomarker, autophagy

## Abstract

**Background:**

The goal of this study was to identify potential predictive biomarkers for the therapeutic effect of infliximab (IFX) in Rheumatoid arthritis (RA) and explore the potential molecular mechanism of nonresponse to IFX treatment to achieve individualized treatment of RA.

**Methods:**

Differential gene expression between IFX responders and nonresponders in the GSE58795 and GSE78068 datasets was identified. Coexpression analysis was used to identify the modules associated with nonresponse to IFX therapy for RA, and enrichment analysis was conducted on module genes. Least absolute shrink and selection operator (LASSO) regression was used to develop a gene signature for predicting the therapeutic effect of IFX in RA, and the area under the receiver operating characteristic curve (AUC) was used to evaluate the predictive value of the signature. Correlation analysis and single-sample gene set enrichment analysis (ssGSEA) were used to explore the potential role of the hub genes. Experimental validation was conducted in synovial tissue and RA fibroblast-like synoviocytes (RA-FLSs).

**Results:**

A total of 46 common genes were obtained among the two datasets. The yellow-green module was identified as the key module associated with nonresponse to IFX therapy for RA. We identified a 25-gene signature in GSE78068, and the AUC for the signature was 0.831 in the internal validation set and 0.924 in the GSE58795 dataset(external validation set). *Derlin-1 (DERL1)* was identified as the hub gene and demonstrated to be involved in the immune response and autophagy regulation. DERL1 expression was increased in RA synovial tissue compared with OA synovial tissue, and DERL1-siRNA partially inhibited autophagosome formation in RA-FLSs.

**Conclusion:**

The 25-gene signature may have potential predictive value for the therapeutic effect of IFX in RA at the beginning of IFX treatment, and autophagy may be involved in nonresponse to IFX treatment. In particular, DERL1 may be associated with the regulation of autophagy.

## Introduction

Rheumatoid arthritis (RA) is a common chronic autoimmune systemic inflammatory disease characterized by chronic inflammatory hyperplasia of the synovium (1). Persistent synovitis could lead to the destruction of joint structure, joint deformity, and loss of joint function (2). RA usually occurs in women around middle age or at the time of menopause, and it can also occur at any age (1). The ratio of males to females is 1:2 to 1:3 and the global average incidence of RA is approximately 0.3% ~ 1% (3). The pathological mechanism of RA is still unclear, and there is still no definitive cure for RA (1, 3).

Tumor necrosis factor-α (TNFα) is an important pathological inflammatory factor in RA that is closely related to synovial inflammation and joint destruction in RA. Clinical studies have shown that anti-TNFα biologic agents can significantly attenuate RA and delay the progression of RA (4). Currently, five anti- TNFα biologic agents are widely used for RA treatment: infliximab (IFX), etanercept (ETN), adalimumab (ADA), certolizumab pegol, and golimumab (5–8).IFX is a chimeric human/mouse monoclonal antibody, in which the Fc domain is of human origin and the Fab’ domain is of mouse origin (4). IFX can specifically bind to biologically active TNFα, inhibit its downstream signal transduction, and plays an anti-inflammatory and immune regulatory role (9). IFX is currently mainly applied to RA, psoriasis, ankylosing spondylitis, ulcerative colitis, Crohn’s disease, and other immune-inflammatory diseases (10). Studies have found that, in the early stage of RA, the combined use of IFX and methotrexate (MTX) can effectively inhibit synovial inflammation and reduce joint destruction, and its efficacy is greater than MTX therapy alone (11). In addition, IFX is also used in patients who do not respond to other anti-TNFα biologic agents, such as ADA or ETN (12). Therefore, IFX is particularly important for the control of RA, especially in the early stage. The introduction of IFX has been proven to be advantageous for many RA patients, but not all patients benefit from IFX treatment. Clinical application shows that there are still some patients who do not respond to IFX (13), and the patients have to increase the IFX dose, which leads to higher side effects and a larger economic burden. At present, the molecular mechanism of nonresponse to IFX treatment for RA is not fully understood. It has been found that the formation of anti-IFX antibodies is one of the causes of nonresponse to IFX treatment in patients with Crohn’s disease and RA (14–17). It is not clear who would benefit from IFX treatment before the treatment begins. Therefore, accurate prediction of IFX efficacy by biomarkers before treatment is helpful to achieve individualized treatment of RA.

Autophagy is a physiological process that is essential for cell growth, differentiation, development, survival and homeostasis (18). It is an adaptive cell response that allows the cell to survive bioenergetic stress such as nutrient deprivation or other stresses (19). It has been found that anti- TNFα biologic agents can induce apoptosis of peripheral blood lymphocytes, and the sensitivity to apoptosis induced by the agents varies widely among RA patients (20). Further research found that the difference in sensitivity to anti- TNFα agents may be related to autophagy state of peripheral blood mononuclear cells (PBMCs). Vomero et al. found that TNFα was able to induce autophagy in RA PBMCs and RA-FLSs in a dose-dependent manner, and PBMCs from patients with RA responsive to anti- TNFα biologic agents showed a reduced autophagy and an increased apoptotic activation (21). These studies suggest that autophagy may be a potential molecular mechanism of nonresponse to IFX therapy in RA.

To understand the early molecular changes and potential molecular mechanisms of nonresponse to IFX therapy in RA, we analyzed the expression profiles of RA patients treated with IFX from the Gene Expression Omnibus (GEO) database. We used common genes in different datasets to establish a least absolute shrink and selection operator (LASSO) model in the training set to develop a gene signature for predicting the therapeutic effect of IFX in RA treatment, and used receiver operating characteristic curve (ROC) to evaluate the predictive value of the model in both internal validation set and external validation set. Weighted gene coexpression network analysis (WGCNA) was used to identify modules associated with nonresponse to IFX therapy in RA, and Gene Ontology (GO) and Kyoto Encyclopedia of Genes and Genomes (KEGG) analyses of module genes were performed. The same genes in the LASSO model and the WGCNA modules were the hub genes of nonresponse to IFX therapy of RA. The bioinformatic analysis was followed by clinical validation. The goal of the present study was to identify predictive biomarkers of the therapeutic effect of IFX in RA treatment and explore the potential molecular mechanism, which was conducive to the individualized treatment of RA.

## Materials and Methods

### Human Tissue Collection and Cell Culture

A total of 9 OA and 9 RA patients’ synovial tissue samples were obtained, and all the patients fulfilled the American College of Rheumatology (ACR) criteria for OA or RA and provided informed consent. This study was approved by the human research ethics committee of Xi’an Hong Hui Hospital (Xi’an Hong Hui Hospital, Xi’an, China). RA fibroblast-like synoviocytes (RA-FLSs) were obtained according to the previous method with some modifications (22). Briefly, RA synovial tissue without adipose tissue and cartilage was washed in sterilized saline and diced thoroughly. Next, the tissue was incubated in trypsin (2.5 mg/mL) (Sigma Co, St. Louis, Missouri, USA) at 37°C for 30 minutes and then removed the trypsin solution. After that, the synovial tissue was then transferred into Type I collagenase solution (2 mg/mL) at 37°C for another 3 hours. The isolated cells were cultured in DMEM/F12 medium (Thermo Fisher Scientific, Waltham, MA, USA) supplemented with 10% fetal bovine serum (Gibco Life Technologies, USA), 100 μg/mL streptomycin, and 100 units/mL penicillin at 37°C under 5% CO_2_. Following the above procedure, 3rd- to 8th- passage RA-FLSs were used for further experiments. The patients’ information is shown in **Table 1**.

**Table 1:** Patient characteristics.

	OA	RA
Patients	9	9
Sex, n, female/male	2/7	6/3
Age^a^, yr	65 (51-75)	59 (55-73)
RF^b^, IU/ml	10.7 ± 4.3	107.5 ± 67.2
Anti-CCP–positive, n	0/9	7/9
ESR^b^, mm/h	15 ± 7.1	43 ± 12.9
CRP^b^, mg/L	3.7 ± 3.5	19.8 ± 18.3
Medications, number of patients		
NSAIDs	3	0
NSAIDs +Steroids+ Chinese herbal medicine	2	2
NSAIDs +Biological agents	0	1
NSAIDs +Steroids+ Chinese herbal medicine +MTX+ Folic acid	0	3
NSAIDs + Chinese herbal medicine +MTX+ Folic acid	0	3
NSAIDs + Chinese herbal medicine	4	0

RF, rheumatoid factor; CCP, cyclic citrullinated peptide; ESR, erythrocyte sedimentation rate; CRP, C-reactive protein; NSAIDs, nonsteroidal anti-inflammatory drugs; MTX, methotrexate.

^a^Median (range), ^b^Mean ± standard deviation.

### Data Sources

We collected data related to IFX treatment for RA from the GEO database. Two datasets were obtained, including the GSE58795 dataset and the GSE78068 dataset, and the gene expression data in both datasets were obtained from baseline whole blood. The GSE58795 dataset included 23 nonresponders and 36 responders, and the GSE78068 dataset included 98 nonresponders and 42 responders. Three datasets GSE77298, GSE55457, and GSE89408, which gene expression data were obtained from synovium, were selected as the validation sets to verify the expression of the hub genes. The GSE77298 dataset included 7 healthy control synovium and 16 RA synovium, the GSE55457 dataset included 10 healthy synovium and 13 RA synovium, and the GSE89408 dataset included 28 healthy synovium and 152 RA synovium. The information for selected datasets is presented in **Table 2**.

**Table 2:** Information for selected datasets.

GEO accession	Samples		Samples		Source tissue	Attribute
	Nonresponders	Responders	Healthy	RA		
GSE78068	98	42	–	–	Whole blood	Training set and internal validation set
GSE58795	23	36	–	–	Whole blood	External validation set
GSE77298	–	–	7	16	Synovium	Validation set
GSE55457	–	–	10	13	Synovium	Validation set
GSE89408	–	–	28	152	Synovium	Validation set

### Identification of Differential Gene Expression

Differentially expressed genes (DEGs) between responders and nonresponders in the GSE58795 and GSE78068 datasets were identified using the ‘Limma’ R package (23), and the genes whose expression differences with a *P* value less than 0.05 were defined as DEGs. All the DEGs were visualized in volcano plots using the ‘ggplot2’ R package, and the common DEGs were visualized in two Venn diagrams using the ‘VennDiagram’ R package.

### Gene Set Variation Analysis

Gene set variation analysis (GSVA) was carried out to identify the differences in the signaling pathways and biological processes between responders and nonresponders in the GSE78068 dataset using the ‘GSVA’ and the ‘Limma’ R packages (24).

### Weighted Gene Coexpression Network Analysis

The top 10000 genes with the lowest *P* values in the GSE78068 dataset were used to identify the coexpression network using the ‘WGCNA’ R package (25). Briefly, after extracting the 10000 genes, the scale-free distribution topology matrix was calculated. The “pickSoflThreshold” function was used to select the optimal soft threshold power (the soft threshold power was set as 9 when the scale-free R^2^ threshold was 0.9 in the present study), and the Pearson correlation coefficient of each gene was calculated. The adjacency matrix was constructed using the weighted Pearson correlation coefficient, and then the adjacency matrix was transformed into a topological overlap matrix (TOM) to construct the hierarchical clustering tree. Different gene modules were identified based on the hierarchical clustering tree. The modules with a gene number greater than 30 were retained, and those with a similarity greater than 0.5 were merged. The modules that were highly correlated with nonresponse to IFX therapy in RA were identified as the key modules.

### Function Enrichment Analysis

GO and KEGG analyses in the key module and the GSE78068 dataset were conducted using the ‘ClusterProfiler’ R package (26). The top three terms of biological processes (BP), components (CC), and molecular functions (MF) with adjusted *p* < 0.05 were visualized using the ‘Goplot’ R package (27), and all KEGG terms with adjusted *p* < 0.05 were visualized using the “ClueGO” plugin in Cytoscape software (28). Gene set enrichment analysis (GSEA) in the GSE78068 dataset was also conducted to explore the KEGG pathways associated with the hub genes using the ‘ClusterProfiler’ R package, and the results with adjusted *p* < 0.05 were considered statistically significant.

### LASSO Regression

Those common DEGs in the same direction in the GSE58795 and GSE78068 datasets were used to construct a LASSO model using the ‘glmnet’ R package. In the GSE78068 dataset, 70% of samples were randomly selected as the training set, and the other 30% of samples were selected as the test set (internal validation set). The samples in the GSE58795 were selected as the validation set (external validation set). ROC curves were constructed to evaluate the predictive value of the key genes using the ‘pROC’ R package (29). The same genes in the LASSO model and the key modules identified by WGCNA were defined as hub genes.

### Single-Sample Gene Set Enrichment Analysis (ssGSEA)

Autophagy-related genes were integrated from the GO_AUTOPHAGY gene set on the GSEA website (http://software.broadinstitute.org/gsea/index.jsp) and in the Human Autophagy Database (HADb, http://www.autophagy.lu/index.html) and used as marker genes of autophagy (30). The enrichment score of autophagy in the GSE78068 dataset was calculated using the ssGSEA function of the ‘GSVA’ R package. The differences of the autophagy score between responders and nonresponders in the GSE78068 dataset were analyzed using Student’s t-test, and the correlations between the autophagy score and the hub genes in nonresponders were also detected using Pearson’s correlation.

### Oligonucleotides and Cell Transfection

DERL1-siRNA targeting human DERL1 and negative control siRNA were chemically synthesized by GenePharma (GenePharma, Shanghai, China). RA-FLSs were plated in 6-well plates at a density of 1.5× 10^5^/well. Twenty-four hours after plating, siRNA (80 nM) or negative control siRNA (80 nM) was transfected into RA-FLSs using Lipofectamine 2000 Transfection Reagent (Invitrogen; Thermo Fisher Scientific, Inc.) according to the manufacturer’s recommendations. The cells were cultured for another 48 hours (for protein) or 24 hours (for mRNA) and then used in the experiments. The siRNA sequence information is given in **Supplementary Table 1**.

### RNA Extraction and Real-Time PCR

Total RNA was isolated from RA and OA synovial tissue and cells using TRIzol reagent (Invitrogen; Thermo Fisher Scientific, Inc.) according to the manufacturer’s protocol. Two micrograms of total RNA was used to synthesize cDNA using a Transcriptor cDNA Synthesis Kit (Thermo Fisher Scientific, Waltham, MA, USA), followed by real-time PCR to detect mRNA expression using a SYBR Green System (Roche Diagnostics, Mannheim, Germany). β-actin expression was used as an endogenous control to normalize the target gene mRNA expression. The 2−ΔΔCT method was used to quantify the relative expression. The primer sequence information is listed in **Supplementary Table 1**.

### Western Blotting

Twenty micrograms of protein sample was separated by 12% sodium dodecyl sulfate-polyacrylamide gel electrophoresis (SDS-PAGE), and then transferred to nitrocellulose membranes (Millipore, Billerica, MA, USA). After blocking in 10% milk in Tris-buffered saline/Tween-20 (TBST) for 2 h at room temperature, the nitrocellulose membranes were incubated with the following primary antibodies overnight at 4°C: anti-LC3 monoclonal antibody (Sigma-Aldrich, St. Louis, MO, USA, 1: 1000), anti-P62 monoclonal antibody (Abcam, Cambridge, UK, 1:10000), anti-β-actin monoclonal antibody (Bioss, Beijing, China, 1:1000), and anti-DERL1 polyclonal antibody (Abcam, Cambridge, UK, 1:2000). The membranes were then incubated with goat anti-rabbit horseradish peroxidase-conjugated secondary antibodies (BOSTER, Wuhan, China, 1:10000) for 2 h at room temperature, followed by expression detection with an electrochemiluminescence (ECL) system (Gene Gnome 5, Synoptics Ltd., UK).

### Immunohistochemistry

After the RA and OA synovial tissue histologic slices were dewaxed, 3% H_2_O_2_ was used to inhibit endogenous peroxide, and microwave heating was used to retrieve antigen. The slices were blocked with 5% goat serum (Beyotime Institute of Biotechnology, Shanghai, China) for 30 minutes, and then incubated in DERL1 antibodies (1:200) overnight at 4°C, and 5% bovine serum albumin (BSA) was used as the negative control. The histologic slices were then incubated with a horseradish peroxidase-conjugated secondary antibody (BOSTER, Wuhan, China) for 30 minutes, followed by staining with 3,3’- diaminobenzidine (Beyotime Institute of Biotechnology, Shanghai, China) and mounting. Then, the DERL1-positive cells (brown cells) was examined using a microscope (Olympus, Tokyo, Japan).

### Autophagic Flux Detection

RA-FLSs were plated in 6-well plates at a density of 1.5× 10^5^/well. Twelve hours after plating, the RA-FLSs were transfected with DERL1-siRNA (80 nM) or negative control siRNA (80 nM) using Lipofectamine 2000 according to the manufacturer’s recommendations. Cells were then cultured for another 48 hours, and 0.1 μM of bafilomycin A1 (Baf-A1) (Sigma-Aldrich, St. Louis, MO, USA) or matched amounts of dimethyl sulfoxide (DMSO) were added to cell cultures for the last 4 hours, and then detected the expression of LC3B, P62 and DERL1 with western blotting.

### mCherry-GFP-LC3B Adenovirus Transfection

RA-FLSs were plated in 12-well plates at a density of 5× 10^4^/well. Twelve hours after plating, the mCherry-GFP-LC3B adenovirus (Beyotime Institute of Biotechnology, Shanghai, China) at 20 multiplicity of infection (MOI) was added to the cells according to the manufacturer’s protocol. Twenty-four hours after infection, the RA-FLSs were transfected with DERL1-siRNA (80 nM) or negative control siRNA (80 nM) using Lipofectamine 2000 according to the manufacturer’s recommendations. Cells were then cultured for another 48 hours, and 0.1 μM of bafilomycin A1 (Sigma-Aldrich, St. Louis, MO, USA) or matched amounts of dimethyl sulfoxide (DMSO) were added to cell cultures for the last 4 hours, and then photographed under a fluorescence microscope (Leica, Germany).

### Statistical Analysis

The data of the experimental validation part are presented as the mean ± standard deviation (SD) or mean with 95% confidence interval (CI). If the data were normally distributed, Student’s T-test was used to analyze the differences between two groups, whereas the Mann-Whitney U test was used. GraphPad Prism 5.0 software (GraphPad Software, La Jolla, CA, USA) was used for data analysis, and *P* values less than 0.05 were considered statistically significant.

## Results

### Identification of Differentially Expressed Genes

**Figure 1:**
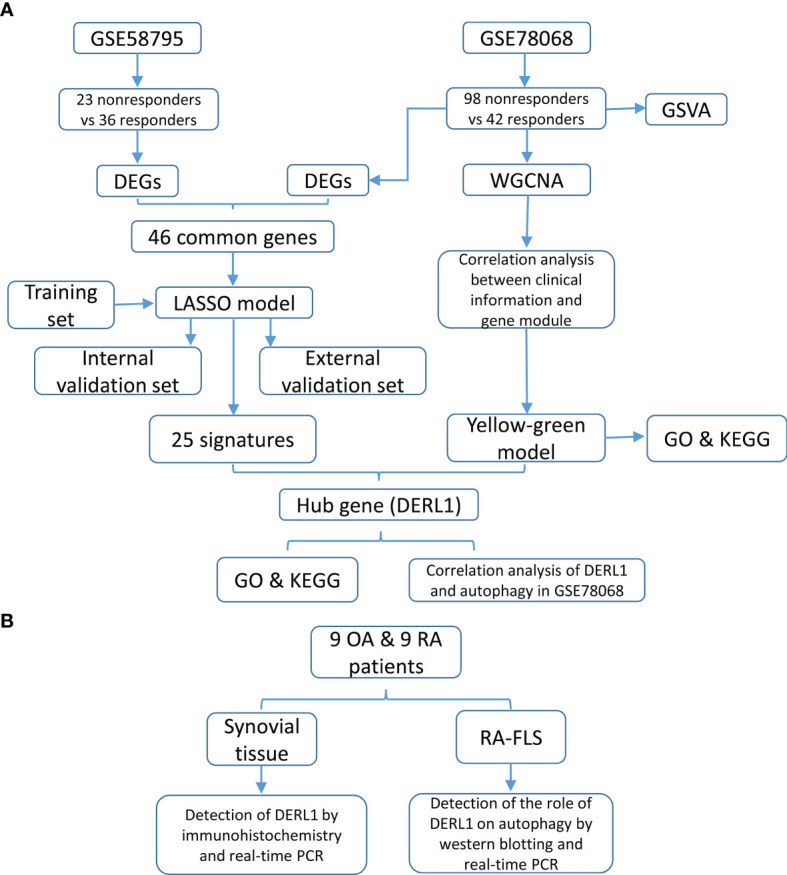
Study design and flowchart. **(A)** The flowchart of construction of predictive model and identification of the hub genes. **(B)** The flowchart of experimental validation of the hub genes.

**Figure 2:**
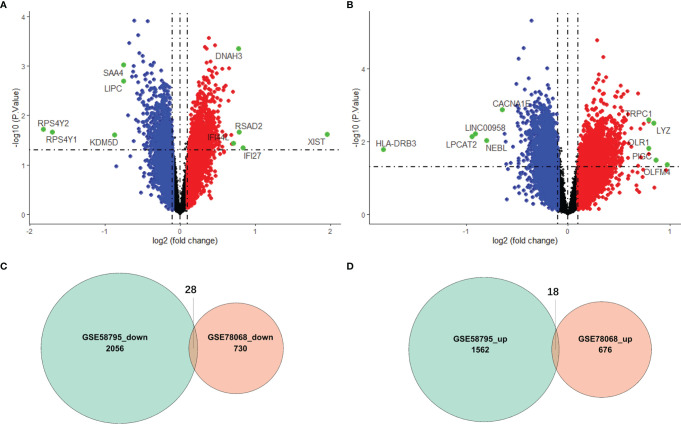
Identification of differentially expressed genes (DEGs). **(A)** Volcano plot of DEGs between responders and nonresponders in IFX treatment for RA in the GSE78068 dataset. **(B)** Volcano plot of DEGs between responders and nonresponders in IFX treatment for RA in the GSE58795 dataset. **(C)** The Venn diagram of the concordant downregulated genes in the two datases. **(D)** The Venn diagram of the concordant upregulated genes in the two datases.

The flowchart is shown in **Figure 1**. To identify the DEGs between responders and nonresponders treated with IFX in RA, we downloaded datasets related to IFX treatment for RA from the GEO database. A total of two datasets were obtained. In the GSE78068 dataset, there were 98 nonresponders and 42 responders, and a total of 1452 DEGs (*p* < 0.05) were identified (**Figure 2A**). In the GSE58795 dataset, there were 23 nonresponders and 36 responders, and a total of 3664 DEGs (*p* < 0.05) were identified (**Figure 2B**). Among these DEGs, 46 genes were common DEGs, including 28 concordant downregulated genes and 18 concordant upregulated genes(**Figures 2C, D** **)**. The 46 common genes were chosen for subsequent analysis.

**Figure 3:**
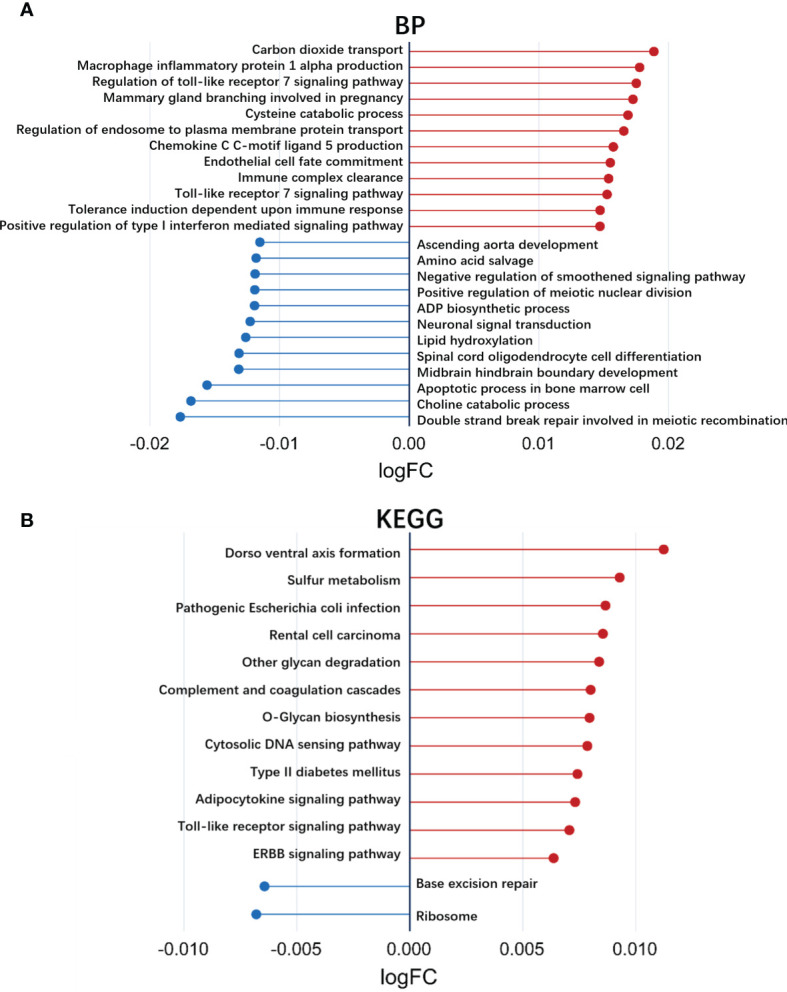
Exploration of the different biological processes terms and KEGG pathways using gene set variation analysis (GSVA). **(A)** Up- or down-regulated biological processes between responders and non-responders in IFX treatment for RA in the GSE78068 dataset quantified by GSVA. **(B)** Up- or down-regulated KEGG pathways between responders and non-responders in IFX treatment for RA in the GSE78068 dataset quantified by GSVA.

We also used GSVA to explore the different BP terms and KEGG pathways between responders and nonresponders in the GSE78068 dataset. Several different BP terms (*P*<0.05) relevant to immune and inflammatory responses and intracellular substance metabolism and transport were mainly enriched in nonresponders (**Figure 3A**, **Supplementary Table 2**). For the KEGG pathway, nonresponders were positively correlated with sulfur metabolism, complement and coagulation cascades, cytosolic and sensing pathways, adipocytokine signaling pathways, Toll-like receptor signaling pathways, receptor tyrosine kinase (ERBB) signaling pathways and so on (**Figure 3B**, **Supplementary Table 3**), which might be related to nonresponse to IFX therapy for RA.

### Identification of the Key Modules Associated With Nonresponse to IFX Therapy for RA

**Figure 4:**
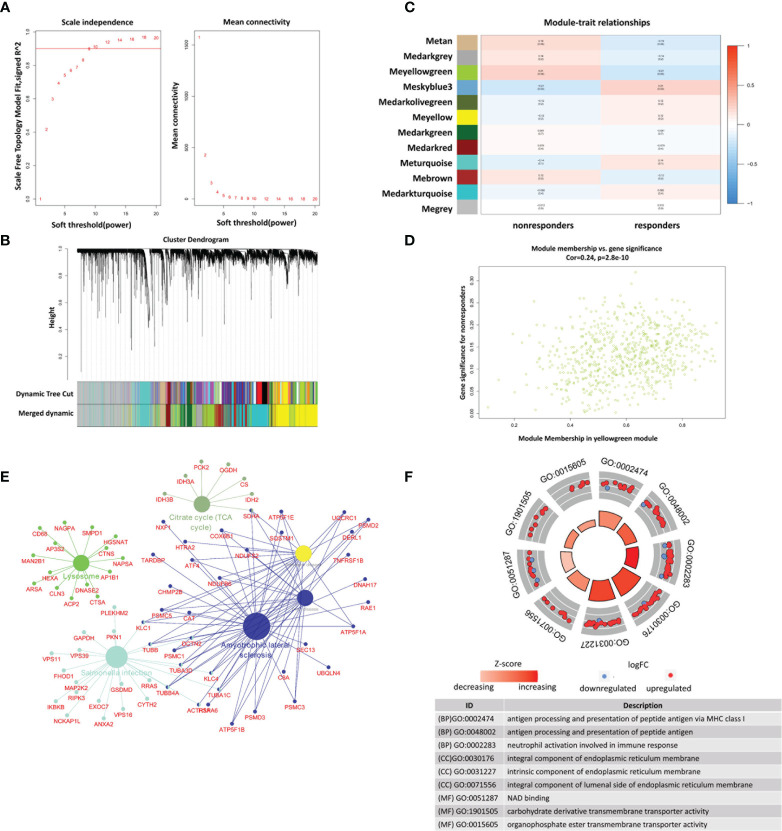
Identification of modules associated with nonresponse to IFX treatment in RA in GSE78068 dataset through weighted gene coexpression network analysis (WGCNA). **(A)** Correlation between scale free topology model fit and soft threshold power. **(B)** The cluster dendrogram showing the change of modules before and after merging, and different colors represent different modules. **(C)** Module-trait relationships. Each row represents a module and each column represents a clinical trait (responders and nonresponders). **(D)** Scatter plot of genes in the yellow-green module. **(E)** The network of KEGG pathway in the yellow-green module. **(F)** The GO analysis of the genes in yellow-green module.

To identify the key modules associated with nonresponse to IFX therapy for RA in the GSE78068 dataset, we performed WGCNA. By setting the optimal soft power threshold for WGCNA as 9 and the cut height as 0.5, scale-free topology is preserved (**Figure 4A**). Twelve modules were identified (**Figures 4B, C** **)**. The heat map of module–trait relationships showed that the yellow-green module was most associated with nonresponse to IFX therapy for RA (correlation coefficient = 0.21, *p* = 0.04), and there were 674 genes in the yellow-green module. The correlation between module membership (MM) in the yellow-green module and gene significance (GS) for nonresponders was also performed, and the results showed that the yellow-green module was highly correlated with nonresponders (correlation coefficient = 0.24, p = 2.8e–10, **Figure 4D**). We also performed GO function and KEGG pathway enrichment analysis with the genes in yellow-green module. The results showed that 147 BP, 47 CC, and eight MF GO terms were enriched, and the genes were mainly enriched in processes such as antigen processing and presentation of peptide antigen *via* MHC class I in BP, integral component of endoplasmic reticulum membrane in CC, and carbohydrate derivative transmembrane transporter activity in MF (**Figure 4F**). For KEGG pathway analysis, six pathways were enriched, and the genes of the six pathways and their connectivity are shown in **Figure 4E**.

### Construction of the Prediction Model and Identification of the Hub Genes Associated With Nonresponse to IFX Therapy for RA

**Figure 5:**
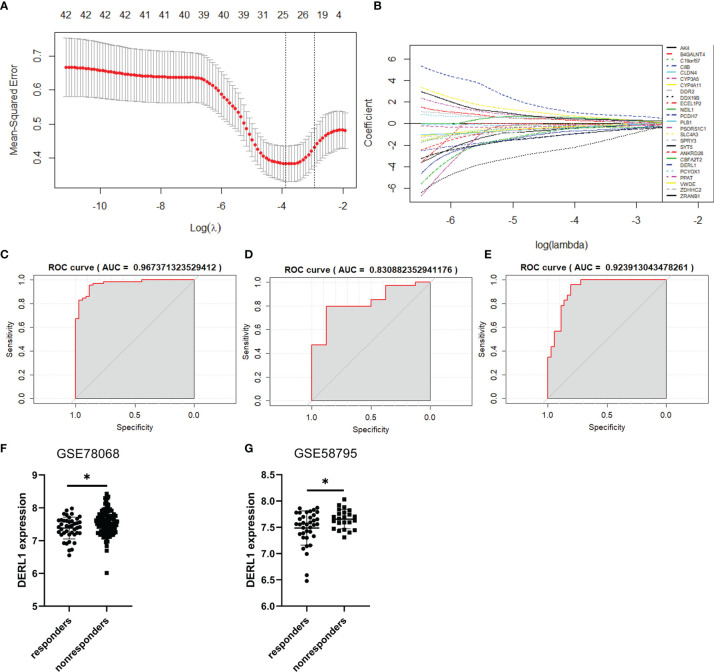
Construction of the prediction model and identification of the hub genes associated with nonresponse to IFX therapy for RA. **(A)** Cross-validation for the gene signature selection of optimal lambda value in least absolute shrink and selection operator (LASSO) model. **(B)** LASSO coefficient profiles of the 25 prediction genes. **(C)** The receiver operating characteristic (ROC) curves of the 25-gene signature in training set of GSE78068 dataset. **(D)** The ROC curves of the 25-gene signature in internal validation set of GSE78068 dataset. **(E)** The ROC curves of the 25-gene signature in external validation set of GSE58795 dataset. **(F, G)** Differential expression of DERL1 between responders and nonresponders in GSE78068 and GSE58795 datasets. Student’s-t test was used for the data analyses in **(F)**. Student’s-t test with Welch’s correction was used for the data analyses in **(G)**. *P < 0.05.

We constructed a LASSO model to identify the key genes associated with nonresponse to IFX therapy for RA. The samples in the GSE78068 dataset were randomly split into a training set (70%) and an internal validation set (30%). The 46 common genes identified above were used to construct a LASSO model in the training set. The LASSO results showed that when the lambda value was selected as lambda.min (0.0207), a total of 25 genes with nonzero coefficients were screened out (**Figures 5A, B**, **Supplementary Table 4**). The AUCs for the 25-gene signature were 0.967 and 0.831 in the training set and the internal validation set, respectively (**Figures 5C, D** **)**. We also validated the signature using the external validation set and GSE58795 dataset, and the AUC was 0.924 (**Figure 5E**). The results showed that the 25-gene signature had good diagnostic ability.

The common genes in the LASSO model and the yellow-green module identified by WGCNA were further screened. Interestingly, only one gene, *DERL1*, was screened out, so *DERL1* was recognized as a hub gene for subsequent validation and analysis. The expression of DERL1 was increased in nonresponders in both the GSE78068 and GSE58795 datasets (**Figures 5F, G** **)**.

### Potential Functions of DERL1 in Nonresponse to IFX Therapy for RA

**Figure 6:**
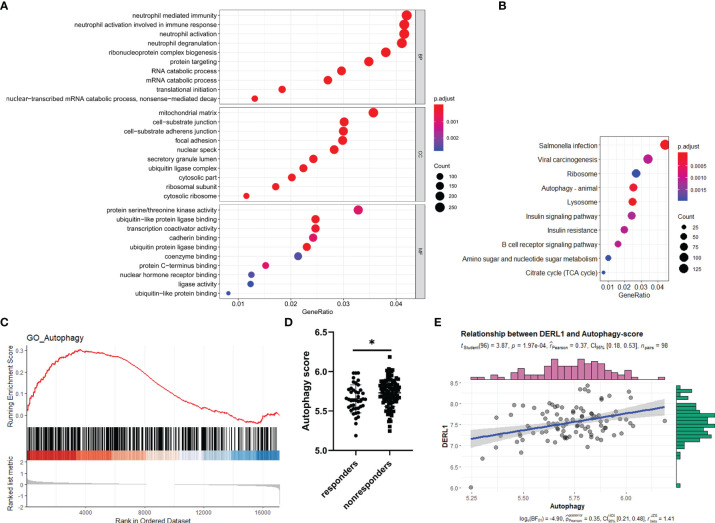
Potential role of DERL1 in nonresponse to IFX therapy for RA. **(A, B)** The GO and KEGG analysis of the genes screened out through a batch correlation analysis between DERL1 and other genes in nonresponders of GSE78068 dataset. **(C)** Gene set enrichment analysis (GSEA) for comparing autophagy gene term between responders and nonresponders in GSE78068 dataset. **(D)** Differential autophagy score between responders and nonresponders in GSE78068 dataset. **(E)** Scatter plot of the relationship between DERL1 expression and autophagy score in nonresponders of GSE78068 dataset. Student’s-t test was used for the data analyses in **(D)**. *P < 0.05.

To gain further insight into the potential role of *DRL1* in nonresponse to IFX therapy for RA, we first conducted a batch correlation analysis between *DRL1* and other genes in nonresponders of the GSE78068 dataset. A total of 8366 genes (*p* < 0.05) were screened for subsequent gene enrichment analysis. GO and KEGG analyses were performed by the clusterProfiler package. BP was mainly enriched in neutrophil immune responses, such as neutrophil-mediated immunity, neutrophil activation involved in the immune response, and neutrophil activation. The cellular component (CC) results revealed that these genes were mainly enriched in the mitochondrial matrix, cell substrate adherens junctions, focal adhesion and so on. For the MF results, the genes were mainly involved in protein serine/threonine kinase activity, ubiquitin-like protein ligase binding, and transcription coactivator activity (**Figure 6A**). The KEGG analysis revealed that these genes were enriched in pathways mainly related to autophagy, lysosome, and B cell receptor signaling pathways (**Figure 6B**). Interestingly, both the BP and KEGG analysis results showed that these genes were associated with autophagy (**Supplementary Table 5, Table 6**), which indicated that there was a relationship between DERL1 and autophagy.

### Autophagy Might Play a Role in Nonresponse to IFX Therapy for RA

From the study above, *DERL1* was identified as the key gene associated with nonresponse to IFX therapy for RA, and we also found that there was a potential role of DERL1 in autophagy. Based on these results, we speculated that autophagy might also play a role in nonresponse to IFX therapy for RA. To identify the difference of the autophagy-related BP between responders and nonresponders in the GSE78068 dataset, we performed GSEA. Autophagy-related genes were integrated from the GO_AUTOPHAGY gene set in the GSEA website and the Human Autophagy Database (HADb), and the GSEA results showed that autophagy-related genes were mainly enriched in nonresponders (**Figure 6C**). Moreover, we used the above autophagy-related genes to analyze the autophagy score of the GSE78068 dataset using the ssGSEA method, and the autophagy score was significantly increased in the nonresponders (**Figure 6D**). We also performed a correlation analysis between DERL1 expression and autophagy score. The scatter plot results revealed that DERL1 expression was positively correlated with the autophagy score (**Figure 6E**).

### Experimental Validation of the Relationship Between DERL1 and Autophagy

**Figure 7:**
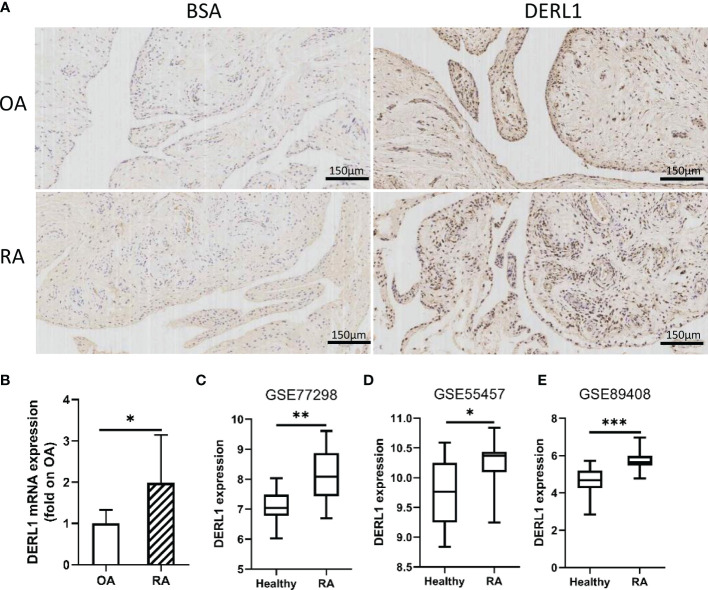
The expression of DERL1 in OA and RA synovial tissues. **(A)** The expression of DERL1 in RA and OA synovial tissue was detected with immunohistochemistry tests. **(B)** The mRNA of DERL1 in RA and OA synovial tissue was detected with real-time PCR. **(C–E)** The expression of DERL1 in GSE77298 dataset, GSE55457 dataset, and GSE89408 dataset. Student’s-t test with Welch’s correction was used for the data analyses in **(B)**. Student’s-t test was used for the data analyses in **(C, D)**. The Mann-Whitney test was used for the data analyses in **(E)**. *P < 0.05, **P < 0.01, ***P < 0.001.

**Figure 8:**
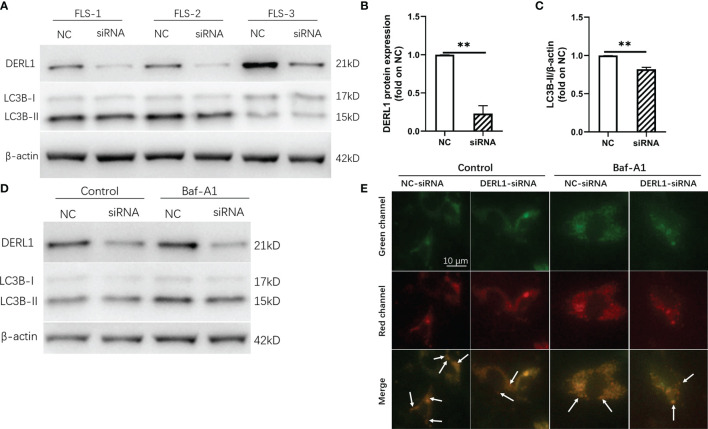
Experimental validation of the relationship between DERL1 and autophagy. **(A)** RA-FLS were transfected with NC-siRNA (80nM) and DERL1-siRNA(80nM) for 48h, the level of DERL1 and LC3B were detected by western blotting. **(B, C)** Relative densitometric analyses of DERL1 and LC3B in **(A)**. **(D)** Autophagic flux was monitored in RA-FLSs after 48 hours of transfection with DERL1-siRNA or NC-siRNA in the presence or absence of 0.1 μM bafilomycin A1 (Baf-A1). **(E)** RA-FLSs infected with mCherry-GFP-LC3B adenovirus were transfected with DERL1-siRNA or NC-siRNA for 48 hours in the presence or absence of 0.1 μM Baf-A1, and the autophagic flux was assessed under fluorescence microscope. Student’s-t test with Welch’s correction was used for the data analyses in **(B, C)**. **P < 0.01.

Chronic inflammatory hyperplasia of the synovium was the most characteristic pathological change in RA. Studies have found that hyperplastic synoviocytes are mainly RA-FLSs, which play a key role in the pathological mechanism of RA. To further validate the expression of DERL1 in RA synovial tissue, we performed real-time PCR and immunohistochemistry. Compared with OA synovial tissue, DERL1 expression was increased in RA synovial tissue (**Figures 7A, B** **)**. We also analyzed DERL1 expression in GSE77298, GSE55457, and GSE89408 dataset, and found that DERL1 expression was increased in RA synovial tissue compared with healthy synovial tissue (**Figures 7C–E**). Furthermore, we explored the effect of DERL1 on the autophagy of RA-FLSs. We used DERL1-siRNA to knockdown DERL1 expression and detected the autophagy-related proteins LC3B. After transfection with DERL1-siRNA, the expression of DERL1 and LC3B-II were decreased significantly (**Figures 8A–C**). The decrease in LC3-II reflects only the decrease of autophagosomes formed, no information about the autophagic flux is provided. Therefore, we transfected DERL1-siRNA in the presence and absence of the lysosomal inhibitor Baf-A1 to block the autophagic pathway at a late stage (31). The results showed that DERL1-siRNA inhibited autophagy formation in RA-FLSs, as demonstrated by an decreased amount of LC3B-II in the presence of Baf-A1 (**Figure 8D**). We also detected the expression of autophagic flux-related protein P62, but no significant changes were found (**Supplementary Figure 1**). The effect of DERL1-siRNA on autophagic flux was further assessed by using mCherry-GFP-LC3B adenovirus. Upon infection of mCherry-GFP-LC3B adenovirus, autophagosomes are labeled with orange or yellow signals, due to both GFP and mCherry fluorescence. However, once autophagosome and lysosome are fused, the autolysosomes will labeled with red puncta, due to GFP fluorescence will be rapidly quenched under low PH of lysosome. As shown in **Figure 8E**, there were fewer orange puncta in the DERL1-siRNA group than in the NC-siRNA group, indicating that there were fewer autophagosomes in the DERL1-siRNA group. When the autophagic flux was blocked by Baf-A1, more orange or yellow puncta were observed in both NC-siRNA and DERL1-siRNA group, and the number of orange or yellow puncta in NC-siRNA group was significantly higher than that in DERL1-siRNA group, indicating that DERL1-siRNA inhibited autophagosomes formation. Based on these results, we speculated that DERL1-siRNA partially inhibited the formation of autophagosomes in RA-FLSs.

## Discussion

Clinical studies have shown that IFX is particularly important for controlling the condition of RA, especially in the early stage. However, the clinical application has found that there are still some RA patients who do not respond to IFX, and the patients have to increase the dose or change the medication in the future. Who would benefit from IFX treatment is still unclear. Therefore, prediction of efficacy through biomarkers before IFX treatment for RA is helpful for individualized treatment of RA. In the present study, we identified a 25-gene signature using the LASSO model and AUCs, and the signature had good predictive ability in both the internal validation set and external validation set. Moreover, *DERL1* was identified as the hub gene, which may be a biomarker to predict the efficacy of IFX in RA treatment. Functional analysis revealed that there was a close relationship between DERL1 and autophagy, and the autophagy score in nonresponders was also increased compared with that in responders to IFX treatment for RA. Finally, the experimental results validated that DERL1 was increased in RA synovial tissues compared with OA synovial tissues and that DERL1-siRNA partially inhibited autophagosomes formation in RA-FLSs.

GSVA is a gene set enrichment (GSE) method that can be used to detect the differences in pathway activity over a sample population in an unsupervised manner (24). In our study, we used GSVA to explore the different BP and KEGG pathways between responders and nonresponders and found that several BP terms associated with inflammation and immunity or intracellular substance metabolism and transport were highly expressed in nonresponders, such as macrophage inflammatory protein 1 alpha production, regulation of Toll-like receptor-7 signaling pathway, and regulation of endosome to plasma membrane protein transport. For the KEGG pathways, nonresponders were positively correlated with the Toll-like receptor signaling pathway and receptor tyrosine kinase (ERBB) signaling pathway. Consistent with Nakamura’s studies (32), they found that inflammasome genes were significantly upregulated with IFX in nonresponders.

Using WGCNA to identify disease-associated modules and explore functional pathways and candidate biomarkers has been proven to be an effective method (25). Twelve modules were identified in the present study, and the yellow-green module was identified as the model most associated with nonresponse to IFX therapy for RA. Consistent with the GSVA results, the enrichment results of the yellow-green module showed that the BP, CC, and MF terms were mainly associated with inflammation and immunity or intracellular substance metabolism and transport. Interestingly, the enriched KEGG pathways were inconsistent with the GSVA results.

We identified a 25-gene signature using a combination of the LASSO model and AUC in our study, and the AUC results suggested that the 25-gene signature was an effective model for the prediction of the efficacy of IFX in RA. Some of the 25 biomarkers, such as phospholipase B1 (PLB1) (33), discoidin receptor 2 (DDR2) (34), and psoriasis susceptibility 1 candidate 1(PSORS1C1) (35), have been identified as biomarkers and participate in the pathogenesis of RA. PSORS1C1 was also related to the efficacy of TNF-α inhibitors in RA treatment. Ciccacci et al. found that SNPs of PSORS1C1 were associated with a severe disease phenotype and response to TNF-α treatment in RA patients (36, 37).

We further identified the hub genes associated with nonresponse to IFX therapy for RA using a combination of the LASSO model and WGCNA and found that only DERL1 was screened out. DERL1, an endoplasmic reticulum (ER)-associated protein, mediates the elimination of misfolded proteins from the ER and retrotranslocation of proteins into the cytosol (38, 39). Studies have reported that the expression of DERL1 is increased in some diseases and it can reduce ER stress-induced apoptosis (40, 41). Dong et al. found that increased DERL1 was correlated with chemoradiotherapy resistance in esophageal squamous cell carcinoma, and the PI3K/AKT/Bcl-2 signaling pathway was involved in this process (42). Interestingly, different results were found in another study (43). Human breast cancer resistance protein (BCRP) could protect cells or tissues from xenobiotic-induced toxicity by facilitating the disposition of endogenous and exogenous harmful xenobiotics, and overexpression of BCRP reduced the intracellular concentration of anticancer drugs. Sugiyama et al. found that DERL1 was involved in the posttranslational regulation of BCRP and was a negative regulator of BCRP expression (43).

To further explore the role of DERL1 in RA, we conducted a batch correlation analysis and ssGSEA in the GSE78068 dataset. The results showed that autophagy-related genes were mainly enriched in nonresponders and that the autophagy score was significantly increased in the nonresponders, which indicated that autophagy might play a role in the nonresponse to IFX therapy for RA and that DERL1 might be involved in autophagy regulation in this process. Studies have found that enhanced autophagy may be associated with the development of drug resistance (44). Our previous study found that the levels of autophagy in RA synovial tissue were increased, MTX stimulated the autophagy response in RA-FLSs, and MTX-induced apoptosis of RA-FLSs was increased after inhibition of autophagy, which indicated that autophagy induction contributed to resistance to MTX treatment in RA-FLSs (45). Xu et al. found that DERL1 expression was elevated in most non-small lung cancer cell lines, and DERL1-siRNA blocked autophagic flux in A549 cells (46). Consistent with the above study, we found that the expression of DERL1 was increased in RA synovial tissue compared with OA synovial tissue. After transfection with DERL1-siRNA, we found that LC3-II expression was decreased, which may be a result of either reduction of autophagosomes formation or increase of autophagic degradation. Therefore, autophagic flux should be detected (31). We found that DERL1-siRNA decreased the expression of LC3-II in the presence of Baf-A1, a lysosomal protease inhibitor, which indicated that DERL1-siRNA might partially inhibit autophagosomes formation in RA-FLSs.

There are still some potential limitations that need to be taken into account when interpreting these findings. First, the two datasets in our study contain different populations of RA; patients in the GSE78068 dataset are Asian, and those in the GSE58795 dataset are European. Second, therapeutic outcomes were defined for the GSE78068 dataset at 6 months after initiation of IFX treatment and the GSE58795 dataset at 14 weeks, which may affect the interpretation of the results. Third, the two datasets were obtained from public databases, and the sample size was still insufficient. Further research is still needed to support our findings. Fourth, we only detected the expression of DERL1 in RA synovial tissue, and its expression in the peripheral blood of RA patients who do not respond to IFX therapy still needs to be detected. More importantly, all synovial tissues were obtained from RA patients who underwent arthroplasty, which means that all RA patients were in the advanced stage of RA. Last, the detailed role of DERL1 in the pathogenesis of nonresponse to IFX therapy for RA remains to be further verified *in vivo* and *in vitro* experiments.

## Conclusion

In conclusion, our 25-gene signature may have potential predictive value for IFX therapy in RA at the beginning of IFX treatment. The results suggest that the autophagy level is increased in nonresponders, which may be involved in nonresponse to IFX therapy for RA. We screened *DERL1* as the hub gene and observed that it was increased in both peripheral blood cells and RA synovial tissue. Moreover, DERL1 is involved in autophagy regulation, and DERL1-siRNA may partially inhibit autophagosomes formation in RA-FLSs. These findings deepen our understanding of the potential molecular mechanism of nonresponse to IFX therapy for RA and provide a direction for future research.

## Data Availability Statement

The datasets presented in this study are available online, and the name of the database and accession number(s) can be found in the article. Further inquiries can be directed to the corresponding author.

## Ethics Statement

The studies involving human participants were reviewed and approved by The human research ethics committee of Xi’an Hong Hui Hospital. The patients/participants provided their written informed consent to participate in this study.

## Author Contributions

PX, YC, and KX designed the study. YC, YA, and ZL carried out the bioinformatic analysis. QY, JX, HZ, and MY conducted the experimental validation. BW and YNY drew the figures. YC, KX, and YA drafted the manuscript. YY and PX participated in modifying the manuscript. All authors approved the final submitted manuscript.

## Funding

This study was supported by the China Postdoctoral Science Foundation (No. 2020M673454), the Foundation of the Natural Science Basic Research of Shaanxi Province of China (2021JQ-924), the Fundamental Research Funds for the Central Universities (xzy012021078), and the National Natural Science Foundation of China (No. 82072432).

## Conflict of Interest

The authors declare that the research was conducted in the absence of any commercial or financial relationships that could be construed as a potential conflict of interest.

## Publisher’s Note

All claims expressed in this article are solely those of the authors and do not necessarily represent those of their affiliated organizations, or those of the publisher, the editors and the reviewers. Any product that may be evaluated in this article, or claim that may be made by its manufacturer, is not guaranteed or endorsed by the publisher.
