# Supplementary material for: Derlin-1, as a Potential Early Predictive Biomarker for Nonresponse to Infliximab Treatment in Rheumatoid Arthritis, Is Related to Autophagy
Source: Front Immunol. 2022 Jan 3;12:795912. doi: 10.3389/fimmu.2021.795912 (PMC8762214; doi:10.3389/fimmu.2021.795912)
Supplement: Supplementary file 1 [file DataSheet_1.pdf]

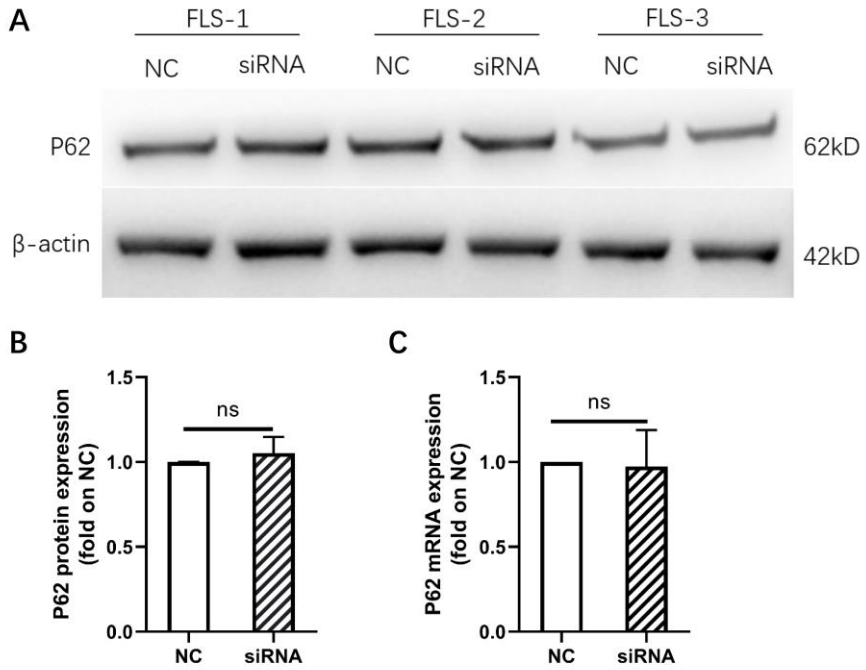

Supplementary figure1 Experimental validation of the relationship between DERL1 and autophagy.

(**A**) RA-FLS were transfected with NC-siRNA (80nM) and DERL1-siRNA(80nM) for 48h, the level of P62 detected by western blotting. (**B**) Relative densitometric analyses of P62 in (**A**). (**C**) P62 mRNA was detected with real-time PCR. Student's-t test with Welch's correction was used for the data analyses. ns, no significantly. The same β-actin with main text was used in here, because the results come from a same detection.
