# Supplementary material for: Derlin-1, as a Potential Early Predictive Biomarker for Nonresponse to Infliximab Treatment in Rheumatoid Arthritis, Is Related to Autophagy
Source: Front Immunol. 2022 Jan 3;12:795912. doi: 10.3389/fimmu.2021.795912 (PMC8762214; doi:10.3389/fimmu.2021.795912)
Supplement: Supplementary file 2 [file Table_1.docx]

Table 1 Sequences of Oligonucleotides

| Name | Sense Strand/Sense Primer(5’-3’) | Antisense Strand/Antisense Primer(5’-3’) |
| --- | --- | --- |
| Primer for real-time PCR |  |  |
| β-actin | ATCGTGCGTGACATTAAGGAGAAG | AGGAAGGAAGGCTGGAAGAGTG |
| DERL1 | CGGACATCGGAGACTGGTTC | AAAAGGTGGCAGTGATTGGC |
| P62 | CAGAGAAGCCCATGGACAG | AGCTGCCTTGTACCCACATC |
| siRNA |  |  |
| NC-siRNA | UUCUCCGAACGUGUCACGUTT | ACGUGACACGUUCGGAGAATT |
| DERL1-siRNA-1 | UGGAUAUGCAGUUGCUGAUTT | AUCAGCAACUGCAUAUCCATT |
| DERL1-siRNA-2 | GAGAGGAGGAGUAUCAGGATT | UCCUGAUACUCCUCCUCUCTT |

Table 2 The different BP terms

|  | logFC | AveExpr | t | P.Value | adj.P.Val | B |
| --- | --- | --- | --- | --- | --- | --- |
| GOBP_CARBON_DIOXIDE_TRANSPORT | 0.018865 | -0.02821 | 2.307506 | 0.022488 | 0.316591 | -5.91818 |
| GOBP_MACROPHAGE_INFLAMMATORY_  PROTEIN_1_ALPHA_PRODUCTION | 0.017765 | 0.036807 | 2.989286 | 0.003302 | 0.302852 | -4.18812 |
| GOBP_REGULATION_OF_TOLL_LIKE_  RECEPTOR_7_SIGNALING_PATHWAY | 0.017506 | 0.178321 | 2.80026 | 0.005826 | 0.302852 | -4.70795 |
| GOBP_MAMMARY_GLAND_BRANCHING_  INVOLVED_IN_PREGNANCY | 0.017251 | -0.21549 | 3.102874 | 0.002317 | 0.302852 | -3.86138 |
| GOBP_CYSTEINE_CATABOLIC_PROCESS | 0.016865 | -0.12262 | 2.083912 | 0.038979 | 0.346739 | -6.39599 |
| GOBP_REGULATION_OF_ENDOSOME_TO_  PLASMA_MEMBRANE_PROTEIN_TRANSPORT | 0.016543 | 0.056565 | 3.152292 | 0.001981 | 0.302852 | -3.71591 |
| GOBP_CHEMOKINE_C_C_MOTIF_LIGAND_  5_PRODUCTION | 0.015735 | 0.014946 | 3.266051 | 0.00137 | 0.302852 | -3.37349 |
| GOBP_ENDOTHELIAL_CELL_FATE_  COMMITMENT | 0.015502 | -0.11622 | 3.11141 | 0.002256 | 0.302852 | -3.83639 |
| GOBP_IMMUNE_COMPLEX_CLEARANCE | 0.01537 | 0.17574 | 2.833287 | 0.005286 | 0.302852 | -4.6193 |
| GOBP_TOLL_LIKE_RECEPTOR_7_  SIGNALING_PATHWAY | 0.015254 | 0.147824 | 2.654571 | 0.008857 | 0.302852 | -5.08785 |
| GOBP_TOLERANCE_INDUCTION_  DEPENDENT_UPON_IMMUNE_RESPONSE | 0.014714 | -0.05133 | 2.41383 | 0.017074 | 0.310146 | -5.67513 |
| GOBP_POSITIVE_REGULATION_OF_TYPE_I_  INTERFERON_MEDIATED_SIGNALING_PATHWAY | 0.014705 | 0.131347 | 3.274713 | 0.001332 | 0.302852 | -3.34699 |
| GOBP_DOUBLE_STRAND_BREAK_REPAIR_  INVOLVED_IN_MEIOTIC_RECOMBINATION | -0.01767 | -0.19421 | -3.15855 | 0.001941 | 0.302852 | -3.69734 |
| GOBP_CHOLINE_CATABOLIC_PROCESS | -0.01685 | -0.19641 | -3.43744 | 0.000773 | 0.302852 | -2.83802 |
| GOBP_APOPTOTIC_PROCESS_IN_BONE_  MARROW_CELL | -0.0156 | -0.10564 | -2.52182 | 0.012791 | 0.305537 | -5.41799 |
| GOBP_MIDBRAIN_HINDBRAIN_  BOUNDARY_DEVELOPMENT | -0.01316 | -0.10575 | -2.57002 | 0.011211 | 0.305537 | -5.2999 |
| GOBP_SPINAL_CORD_OLIGODENDROCYTE_  CELL_DIFFERENTIATION | -0.01313 | -0.23784 | -2.2698 | 0.024742 | 0.320109 | -6.00192 |
| GOBP_LIPID_HYDROXYLATION | -0.01262 | -0.28657 | -2.25296 | 0.025811 | 0.320109 | -6.03892 |
| GOBP_NEURONAL_SIGNAL_  TRANSDUCTION | -0.01227 | 0.001791 | -2.7477 | 0.006789 | 0.302852 | -4.84711 |
| GOBP_ADP_BIOSYNTHETIC_PROCESS | -0.01194 | 0.089253 | -2.04055 | 0.043168 | 0.353993 | -6.48336 |
| GOBP_POSITIVE_REGULATION_OF_  MEIOTIC_NUCLEAR_DIVISION | -0.01192 | -0.1591 | -3.2939 | 0.00125 | 0.302852 | -3.28809 |
| GOBP_NEGATIVE_REGULATION_OF_SMOOTHENED_  SIGNALING_PATHWAY_INVOLVED_  IN_DORSAL_VENTRAL_NEURAL_TUBE_PATTERNING | -0.01189 | -0.1551 | -2.37329 | 0.018984 | 0.311718 | -5.769 |
| GOBP_AMINO_ACID_SALVAGE | -0.01181 | 0.045371 | -2.39105 | 0.018125 | 0.310146 | -5.72807 |
| GOBP_ASCENDING_AORTA_DEVELOPMENT | -0.01152 | -0.09132 | -2.18486 | 0.030557 | 0.330822 | -6.1859 |

Table 3 The different KEGG pathways

|  | logFC | AveExpr | t | P.Value |
| --- | --- | --- | --- | --- |
| KEGG_DORSO_VENTRAL_AXIS_FORMATION | 0.011238 | 0.065836 | 2.616561 | 0.009826 |
| KEGG_SULFUR_METABOLISM | 0.009287 | 0.286093 | 2.17035 | 0.031614 |
| KEGG_PATHOGENIC_ESCHERICHIA_COLI_INFECTION | 0.008659 | 0.404607 | 3.05655 | 0.002668 |
| KEGG_RENAL_CELL_CARCINOMA | 0.008545 | 0.217102 | 2.269976 | 0.024689 |
| KEGG_OTHER_GLYCAN_DEGRADATION | 0.008379 | 0.312566 | 2.068576 | 0.040368 |
| KEGG_COMPLEMENT_AND_COAGULATION_CASCADES | 0.008003 | -0.05286 | 2.048053 | 0.042366 |
| KEGG_O_GLYCAN_BIOSYNTHESIS | 0.007954 | 0.095947 | 2.33416 | 0.020965 |
| KEGG_CYTOSOLIC_DNA_SENSING_PATHWAY | 0.007856 | 0.208031 | 2.804676 | 0.00573 |
| KEGG_TYPE_II_DIABETES_MELLITUS | 0.007418 | 0.044597 | 2.22124 | 0.027891 |
| KEGG_ADIPOCYTOKINE_SIGNALING_PATHWAY | 0.007317 | 0.147107 | 2.58272 | 0.010796 |
| KEGG_TOLL_LIKE_RECEPTOR_SIGNALING_PATHWAY | 0.00706 | 0.21094 | 2.312442 | 0.022166 |
| KEGG_ERBB_SIGNALING_PATHWAY | 0.006363 | 0.102521 | 2.109616 | 0.036616 |
| KEGG_RIBOSOME | -0.00679 | 0.641208 | -2.58679 | 0.010675 |
| KEGG_BASE_EXCISION_REPAIR | -0.00643 | 0.301548 | -2.05709 | 0.041476 |

Table 4 Genes with nonzero coefficients in LASSO model

|  | var_names | coef | expcoef |
| --- | --- | --- | --- |
| 1 | DDX19B | -2.04858 | 0.128917 |
| 2 | C8B | -0.89644 | 0.408018 |
| 3 | SYT5 | -0.87736 | 0.415879 |
| 4 | PCDH7 | -0.65126 | 0.521388 |
| 5 | NEIL1 | -0.40155 | 0.669279 |
| 6 | AK4 | -0.39564 | 0.673251 |
| 7 | PLB1 | -0.33071 | 0.718414 |
| 8 | DDR2 | -0.29868 | 0.741794 |
| 9 | B4GALNT4 | -0.29377 | 0.745452 |
| 10 | SPRY3 | -0.27632 | 0.758571 |
| 11 | CYP4A11 | -0.23232 | 0.792694 |
| 12 | C19orf57 | -0.20601 | 0.813826 |
| 13 | CYP3A5 | -0.10838 | 0.89729 |
| 14 | PSORS1C1 | -0.10737 | 0.898197 |
| 15 | CLDN4 | -0.07156 | 0.930944 |
| 16 | ECEL1P2 | -0.05861 | 0.943071 |
| 17 | SLC4A3 | -0.01178 | 0.988286 |
| 18 | ANKRD28 | 0.272911 | 1.313783 |
| 19 | ZDHHC2 | 0.354335 | 1.425233 |
| 20 | PCYOX1 | 0.412723 | 1.510926 |
| 21 | ZRANB1 | 0.414026 | 1.512896 |
| 22 | PPAT | 0.414182 | 1.513133 |
| 23 | CBFA2T2 | 0.530949 | 1.700545 |
| 24 | VWDE | 0.643862 | 1.903818 |
| 25 | DERL1 | 0.956935 | 2.603704 |
| 26 | (Intercept) | 6.61117 | 743.3523 |

Table 5 The top 15 biological processes (BP) terms

|  | ONTOLOGY | ID | Description | GeneRatio | pvalue | p.adjust |
| --- | --- | --- | --- | --- | --- | --- |
| 1 | BP | GO:0002283 | neutrophil activation involved in immune response | 272/6546 | 1.32E-20 | 8.40E-17 |
| 2 | BP | GO:0043312 | neutrophil degranulation | 269/6546 | 5.43E-20 | 1.46E-16 |
| 3 | BP | GO:0002446 | neutrophil mediated immunity | 275/6546 | 6.90E-20 | 1.46E-16 |
| 4 | BP | GO:0042119 | neutrophil activation | 272/6546 | 5.69E-19 | 9.03E-16 |
| 5 | BP | GO:0006401 | RNA catabolic process | 194/6546 | 1.79E-16 | 2.28E-13 |
| 6 | BP | GO:0000184 | nuclear-transcribed mRNA catabolic process, nonsense-mediated decay | 86/6546 | 5.46E-16 | 5.78E-13 |
| 7 | BP | GO:0006402 | mRNA catabolic process | 177/6546 | 9.69E-16 | 8.79E-13 |
| 8 | BP | GO:0006605 | protein targeting | 228/6546 | 4.23E-15 | 3.36E-12 |
| 9 | BP | GO:0006413 | translational initiation | 120/6546 | 4.49E-14 | 3.17E-11 |
| 10 | BP | GO:0022613 | ribonucleoprotein complex biogenesis | 249/6546 | 2.46E-13 | 1.56E-10 |
| 11 | BP | GO:0000956 | nuclear-transcribed mRNA catabolic process | 123/6546 | 1.30E-12 | 7.50E-10 |
| 12 | BP | GO:0042254 | ribosome biogenesis | 157/6546 | 2.10E-12 | 1.11E-09 |
| 13 | BP | GO:0006914 | Autophagy | 233/6546 | 4.34E-11 | 1.91E-08 |
| 14 | BP | GO:0061919 | process utilizing autophagic mechanism | 233/6546 | 4.34E-11 | 1.91E-08 |
| 15 | BP | GO:0010498 | proteasomal protein catabolic process | 227/6546 | 4.52E-11 | 1.91E-08 |

Table 6 The top 15 KEGG pathways

|  | ID | Description | GeneRatio | pvalue | p.adjust |
| --- | --- | --- | --- | --- | --- |
| 1 | hsa05132 | Salmonella infection | 146/3263 | 2.05E-09 | 6.77E-07 |
| 2 | hsa04142 | Lysosome | 81/3263 | 9.51E-08 | 1.57E-05 |
| 3 | hsa04140 | Autophagy - animal | 82/3263 | 2.31E-06 | 0.000254 |
| 4 | hsa05203 | Viral carcinogenesis | 112/3263 | 1.27E-05 | 0.00105 |
| 5 | hsa04662 | B cell receptor signaling pathway | 52/3263 | 1.72E-05 | 0.001087 |
| 6 | hsa04931 | Insulin resistance | 65/3263 | 1.98E-05 | 0.001087 |
| 7 | hsa04910 | Insulin signaling pathway | 79/3263 | 2.43E-05 | 0.001145 |
| 8 | hsa03010 | Ribosome | 88/3263 | 5.24E-05 | 0.001902 |
| 9 | hsa00020 | Citrate cycle (TCA cycle) | 23/3263 | 5.32E-05 | 0.001902 |
| 10 | hsa00520 | Amino sugar and nucleotide sugar metabolism | 33/3263 | 5.76E-05 | 0.001902 |
| 11 | hsa05171 | Coronavirus disease - COVID-19 | 122/3263 | 7.54E-05 | 0.002262 |
| 12 | hsa04144 | Endocytosis | 131/3263 | 8.31E-05 | 0.002286 |
| 13 | hsa05135 | Yersinia infection | 77/3263 | 0.000101 | 0.002562 |
| 14 | hsa04210 | Apoptosis | 76/3263 | 0.000144 | 0.003388 |
| 15 | hsa04613 | Neutrophil extracellular trap formation | 101/3263 | 0.00018 | 0.003965 |
